# Novel color M-mode echocardiography for non-invasive assessment of the intraventricular pressure in goats: Feasibility, repeatability, and the effect of sedation

**DOI:** 10.3389/fvets.2022.935437

**Published:** 2022-10-06

**Authors:** Ahmed S. Mandour, Haney Samir, Tomohiko Yoshida, Katsuhiro Matsuura, Lina Hamabe, Kazumi Shimada, Hend A. Abdelmageed, Mohamed Elbadawy, Akiko Uemura, Ken Takahashi, Gen Watanabe, Ryou Tanaka

**Affiliations:** ^1^Department of Veterinary Medicine (Internal Medicine), Faculty of Veterinary Medicine, Suez Canal University, Ismailia, Egypt; ^2^Laboratory of Veterinary Surgery, Department of Veterinary Medicine, Tokyo University of Agriculture and Technology, Tokyo, Japan; ^3^Department of Theriogenology, Faculty of Veterinary Medicine, Cairo University, Giza, Egypt; ^4^Ismailia Laboratory, Animal Health Research Institute, Agriculture Research Center, First District, Ismailia, Egypt; ^5^Department of Pharmacology, Faculty of Veterinary Medicine, Benha University, Toukh, Egypt; ^6^Laboratory of Pharmacology, Tokyo University of Agriculture and Technology, Tokyo, Japan; ^7^Division of Veterinary Research, Department of Veterinary Surgery, Obihiro University of Agriculture and Veterinary Medicine, Obihiro, Japan; ^8^Department of Pediatrics and Adolescent Medicine, Juntendo University Graduate School of Medicine, Tokyo, Japan; ^9^Laboratory of Veterinary Physiology, Tokyo University of Agriculture and Technology, Tokyo, Japan

**Keywords:** color M-mode echocardiography, intraventricular pressure, heart function, sedation, feasibility, goats

## Abstract

**Background:**

The intraventricular pressure difference (IVPD) and intraventricular pressure gradients (IVPG), estimated from color M-mode echocardiography (CMME) of the transmitral flow, have been introduced as novel indices for the evaluation of heart functions. Until now, no study demonstrated the feasibility of the CMME approach to measure IVPD and IVPG in any farm animals. The aim of this study was to assess the feasibility and repeatability of CMME-derived IVPD and IVPG variables in goats and explore the effect of sedation on the measured variables.

**Materials and methods:**

Sixteen male Shiba goats were included in this study and underwent conventional echocardiography. Eight goats were used in the repeatability of IVPD/IVPG variables. Another eight goats were used to evaluate the effect of sedation by xylazine on IVPD/IVPG measurements. CMME between the base and the apex of the left ventricle was carried out. The IVPD and IVPG were analyzed using in-house code software. The IVPD and IVPG were expressed as total, basal, mid-to-apical, mid, and apical segments. Data analysis including the imaging quality score (IQS), repeatability, variability, intraclass correlation coefficient (ICC), as well as the effect size of sedation on the measured variables was calculated.

**Results:**

IVPD and IVPG variables from CMME were feasible in all goats. Low to moderate variability of IVPD and IVPG variables was observed (CV 95% <25%) except for the apical IVPD and apical IVPG. The IVPD/IVPG measurements were repeatable without a significant effect of animal or time on the obtained measurements. The overall ICC was higher than 0.75 in all variables except for the apical segment. Xylazine administration reduced the total, basal, and mid parts of IVPD and IVPG with a large effect size (biserial ranked correlation; rc > 0.8).

**Conclusion:**

We reported, for the first time, IVPD and IVPG measurements by CMME in goats. The assessment of IVPD and IVPG by CMME is feasible in goats which can be evaluated in further cardiovascular or pharmacological studies in this species.

## Introduction

Echocardiography is the most common cardiac function evaluation technique utilized in the clinical setting in both human and animal species because of its feasibility and noninvasiveness. The establishment of new echocardiographic techniques for the early detection of cardiac dysfunction is helpful for early interference and better patient outcomes (1). Currently, the assessment of diastolic function is thought to be an important issue for in-depth understanding and early detection of cardiac disorders (2, 3). The diastolic function of the left ventricle (LV) is affected by the heart rate, preload, myocardial relaxation, recoil, and untwist, as well as ventricular compliance and myocardial stiffness. Therefore, the evaluation of diastolic function using traditional echocardiography is somewhat challenging and a combination of various echocardiographic techniques is necessary to avoid the overestimation of the measurements (2–4). Cardiac catheterization is the basic method to diagnose diastolic dysfunction through pressure–volume (PV) analysis and interpretation of the pressure inside the LV, but the technique is invasive and difficult to use in serial studies when longitudinal observation of the same individuals is needed (5).

Recently, the benefit of spatiotemporal maps of the LV hemodynamics has been applied in the context of diastolic function assessment. The velocity pattern along the transmitral scanline can be efficiently evaluated from the analysis of color M-mode echocardiographic (CMME) recordings after processing of images (6, 7) depending principally on Euler's equation (8). By this method, the LV is divided into basal, mid, and apical parts. This segmentation allows for studying the normal stratification of the intraventricular pressure which is known as the intraventricular pressure difference (IVPD, the pressure difference between LV segments), and the intraventricular pressure gradients (IVPG, obtained when the IVPD is divided by the LV length) (9, 10). In early diastole, the negative pressure gradients created by the LV are necessary to restore the LV shape and have an important role in diastolic function evaluation. This pressure gradient comes from the interaction between convective, inertial, and viscous forces in the LV (11).

The IVPD variables derived from CMME have been well-correlated with the same indices measured by the invasive catheterization method (12). Therefore, CMME-derived indices can non-invasively evaluate heart function. Studies revealed that the IVPD and IVPG as novel echocardiographic indices could reflect the diastolic function changes that could differentiate subtypes of heart failure (13). In addition, CMME variables showed more advantages regarding repeatability and continuous data acquisition for better interpretation of the segmental pressure (14–16). To date, the utility of IVPD and IVPG is still under research consideration as a promising tool for the detection of cardiac dysfunction in animal models. Experimental studies revealed that IVPG/IVPD segments could determine the changes in heart function in response to loading states (16), chemotherapy-induced cardiomyopathy (1), LV ventricular hypertrophy (17) in addition to uremic and diabetic cardiomyopathies in animal models (18–20). Other clinical studies were explored IVPD/IVPG in healthy dogs and cats (21, 22), as well as in dogs with patent ductus arteriosus (7, 21, 22).

The anatomical segmentation of the LV in small ruminants was previously reported (23); however, the physiology of intraventricular blood flows in ruminants is still unclear. The assessment of IVPD and IVPG in goats may be important since goats are considered useful candidates in cardiology research because of reasonable heart size and blood flow dynamics (24, 25). They are also regarded as an intermediate model between small animals, large animals, and humans. Besides, goats as a ruminant species can efficiently reflect the physiological, pathological, and pharmacological aspects when used as a model for ruminants (26–29). To date, the evaluation of IVPD/IVPG *via* CMME in any farm animals is lacking. Therefore, this study aimed to evaluate the IVPD and IVPG in goats using CMME. We will describe the technical procedures, and repeatability of measurements, and evaluate the IVPD/IVPG changes after sedation. The results of this study would provide fundamental background on the usability of CMME for the assessment of IVPD/IVPG in goats to refine further cardiovascular research studies in goats or similar species.

## Materials and methods

### Animals

Sixteen male Shiba goats, 2 to 3 years old and weighing 30±5 kg, were enrolled in this study. Animals were kept in a special barn where they received alfalfa hay cubes as a basic diet, while water and mineral blocks were kept *ad libitum*. Detailed physical examination was performed, and the enrolled goats were considered healthy and free from any cardiac abnormalities based on the medical record, cardiac auscultation, routine electrocardiography, echocardiography, and standard hemato-biochemical profile. Two weeks before the study, neither medications nor vaccination was administered. Animals were acclimatized to the hospital to avoid stress-induced erroneous imaging. First, conventional and CMME were performed on eight goats for CMME repeatability. Second, we examined the effect of sedation on heart function through the evaluation of conventional echocardiography and CMME in another eight goats.

### Conventional echocardiography

The echocardiography was carried out from the right and left sides, while goats were maintained on the lateral recumbency position. The right and left precordial areas were prepared with ultrasound gel after shaving. Animals were restrained on the echocardiographic table by two assistants and the forelegs were kept anteriorly. A ProSound Alpha 10 ultrasonography system (Hitachi Aloka Medical, Tokyo, Japan) supplied with a sector probe of 5 MHz was used. A lead II electrocardiogram was arranged and attached to the skin surface to measure the duration of echocardiographic variables. The average of the same echocardiographic variables was obtained from five different consecutive heart cycles by the same operator (A.S.M). The recorded measurements and image orientation were according to the veterinary echocardiography guidelines described by Boon (30).

First, each animal underwent conventional two-dimensional, M-mode, spectral Doppler, and tissue Doppler imaging (TDI) from the standard right and left parasternal views. The right transthoracic long axis four- and five-chamber views were observed. After that, the M-mode short-axis view at the papillary muscle level was viewed to trace the LV measurements which include LV end-diastolic and end-systolic diameters (LVIDd, LVIDs), diastolic and systolic interventricular septal thickness (IVSd, IVSs), diastolic and systolic LV free wall thickness (LVPWd, LVPWs), ejection fraction (EF), and fractional shortening (FS). Aortic root diameter (Ao) was measured at end-diastole and left atrial (LA) dimension was measured at end-systole from the right parasternal short-axis view at the level of heart base, and LA/Ao ratio was calculated. The right ventricular outflow tract (RVOT) was obtained through an assessment of the pulmonary artery using pulsed-wave Doppler echocardiography. The left transthoracic echocardiography was initiated from the left parasternal apical four-chamber view. Aortic blood flow was assessed from the left apical five-chamber view and the the left ventricular outflow tract (LVOT) and cardiac output (CO) were measured. Dual Doppler function of the mitral inflow and tissue Doppler imaging (TDI) was switched on for simultaneous assessment of mitral inflow and tissue Doppler velocity. Diastolic indices including early (E) and late (A) mitral inflow velocities, E/A ratio, and deceleration time were obtained by pulsed-wave Doppler echocardiography. The early and late diastolic myocardial velocities (e′, a′) were measured at both lateral and septal annuli using pulsed TDI. The ratio of early mitral inflow and early tissue velocity (E/e′), as well as annular tissue velocities (e′/a′), were calculated.

### Color M-mode echocardiography

The apical four-chamber view was consistently optimized, which was used to capture CMME images for IVPD/IVPG calculation. First, the mitral inflow was visualized by two-dimensional echocardiography then the IVPG setting of the ultrasound machine was initiated (1). A sweep speed of 300 mm/s and a color baseline shift of −64 were maintained. The M-mode cursor was positioned along the streamline of the transmittal inflow and good quality imaging which showed perfect mitral inflow and mitral valve movement was saved for further analysis. The CMME and image analysis were performed by two observers, once by each observer at a one-day interval (A.S.M and T.Y). Images used in the analysis showed three consecutive heart cycles to ensure consistent mitral inflow and at least five images were saved for analysis by the software. Four images from consecutive heart cycles were selected from each animal to calculate IVPD and IVPG.

### Assessment of the intraventricular pressure

After CMME image acquisition, the IVPD was calculated using in-house code written in MATLAB (The MathWorks, Natick, MA, USA). In each goat, by using the conventional echocardiography images and the displayed ECG, the time from aortic opening to aortic closure (aortic flow images) and the time from Q wave on ECG to the start of mitral valve opening as well as the time from Q wave to peak mitral inflow (mitral inflow images) were obtained and manually inserted into the software during each photo processing by MATLAB (7). Automatic and manual correction of the resulting curves using specific software codes was applied when required to enhance the quality of analysis. The IVPD of each corresponding part was calculated from the following Euler equation.


(∂P)/(∂s)=-ρ((∂v)/(∂t)+v(∂v)/(∂s))

where ∂ is the change in element followed, P is the pressure, ρ is the constant blood density (1,060 kg/m^3^), v is the velocity, s is the position along with the color M-mode line, and t is the time. v, s, and t are obtained by MATLAB which is further used to measure the relative pressures within the region of interest (14, 20, 21). The Euler equation assuming laminar blood flow across the mitral valve and the ultrasound scanline is related to the inflow blood streamline and the IVPD and IVPG can be estimated by solving the equation (12).

Previously, Takahashi and colleagues (31) measure the IVPD and IVPG in young children after the modification of the method used in adult humans (32, 33). Later, the same method was used in dogs and cats (21, 22). We used the same method for IVPD and IVPG calculation. This method assumes the dividing of the LV into three segments (basal, mid, and apical) on the long axis, and the respective IVPD and IVPG were calculated. The data of IVPD obtained from CMME were previously validated against direct measurements using a micromanometer (12). For IVPG calculation, the same image used to assess the IVPD was used to measure the LV length on the long axis, from the level of mitral annulus to the LV apex. The IVPG was calculated by dividing the IVPD by the left ventricular length (34). As previously described (20), the LV was trisected into apical, mid, and basal parts. Mid-to-apical segments of IVPD/IVPG were calculated by adding the mid and apical segments. Both total IVPD and IVPG and their corresponding pressure at the anatomical position in the LV were calculated and expressed as basal IVPD and IVPG; mid IVPD and IVPG; mid-to-apical IVPD and IVPG; and apical IVPD and IVPG (mmHg).

### Sedation of goats

The echocardiography was performed before and after xylazine administration (0.05 mg/kg BW/IM, xylazine hydrochloride, Fujita-Pharm, Japan). Echocardiography was started 10 min after the observation of the signs of xylazine sedation (35). The duration of ultrasonography ranged between 20 and 30 min and no other medication was used during the experiment.

### Statistical analysis

The normality of the data was tested by the Shapiro–Wilk test. The repeatability of IVPD and IVPG variables was assessed by the analysis of variance using two-way ANOVA. Two factors (goat and time), as well as the interaction between goat and time, were considered. The coefficient of variation for each parameter was measured between randomly selected four consecutive cardiac cycles to assess the intraobserver variability and between observers (scans A and B). All measurements from the four heart cycles were pooled from each goat regardless of the examination time to determine the within-goat variability using one-way ANOVA considering only animals as a factor. Moreover, the intraclass correlation coefficient (ICC) was calculated for each variable. The reproducibility of the technique is acceptable when the ICC is equal to or higher than 0.75. Lastly, all images captured from CMME and after CMME image processing by MATLAB were blindly evaluated by another two observers to obtain the imaging quality score (IQS; from zero to five) which was further analyzed using Fisher's exact test. According to the degree of variability, the obtained measurements were classified as low variability (CV <15%), moderate variable (CV% 15–25%), and high variable (CV%> 25%) as previously described (16, 19). The confidence interval of the mean (95% CI) for each variable was calculated to determine the interval within the absolute value which had a 95% of probability being included. CMME variables were considered acceptable if both a non-significant result of the two-way ANOVA and low or moderate variability (95% CV <25%) were observed.

For comparison between normal and sedated goats, the Wilcoxon matched-pairs signed-rank test was calculated between measurements of baseline and after xylazine administration. Analyzed variables showing *p* < 0.05 were considered statistically significant. To quantify the strength of xylazine administration on echocardiographic data, the effect size estimator (Rank-Biserial Correlation, rc) for the Wilcoxon signed-rank test was calculated. Interpretation of the effect size was classified into small, medium, and large depending on cutoffs of 0.1, 0.4, and 0.6, respectively (36). Spearman's rank correlation (r_s_) was expressed as a whole value between measured conventional variables and CMME-derived measurements before and after xylazine injection. The ICC was evaluated by SPSS software version 26.0 (Chicago, IL, USA). The effect size was calculated using a free JASP software version 0.13 (JASP Team, Amsterdam, Netherlands). Other Statistical analyses and graphs were conducted using GraphPad Prism 8.4 (GraphPad Software, San Diego, CA).

## Results

### Conventional echocardiography

The echocardiographic data are presented in Table 1. A significant reduction in HR after sedation compared with the baseline was observed (*P* < 0.05); meanwhile, EF and FS did not show a significant difference. LA/AR, LVOT, and RVOT were slightly increased after xylazine administration. The mitral inflow measurements, as well as the TDI indices, were reduced after xylazine administration. Early (E) and late (A) mitral inflow velocities and deceleration time were significantly reduced, while the E/A ratio did not change. Septal annular wall velocity (a′) and lateral annular velocity (e′) were significantly reduced post-xylazine administration (*P* = 0.02, 0.03, respectively). The ratio of septal e′/a′ was significantly decreased after xylazine administration, while the lateral e′/a′ was reduced but not significant. The LV length was increased after xylazine treatment compared with the baseline (*P* = 0.008). The magnitude of echocardiographic measurements difference between the baseline and post-xylazine administration was clinically relevant and associated with a large effect size on HR, LVOT, E velocity, A velocity, deceleration time, a′ septal, e′/a′ septal, E/e′ septal, and e′ lateral (rc = 0.944, 0.833, 0.778, 1.0, 0.944, 0.833, 0.833, 0.722, 0.611), respectively.

**Table 1:** Conventional echocardiographic measurements before and after xylazine administration in male goats.

**Variables**	**Unit**	**Baseline**	**Post-xylazine**	***P* value**	**Effect size (rc)**
EDV	ml	38.5 (22.5–57)	40 (26–57.5)	0.72	0.167
ESV	ml	10.4 (2.5–14.5)	9.4 (5.1–14.5)	0.30	0.333
EF	%	76.8 (49.9–91.3)	74.2 (60.9–85)	0.71	0.056
FS	%	39.2 (31.9–55.7)	36.45 (27–46.6)	0.09	0.786
LA/Ao		1.55 (1.2–1.7)	1.6 (1.2–2.2)	0.05	0.751
RVOT	mm	15.0 (12.7–16.7)	15.75 (13.5–18.6)	0.17	0.556
LVOT	cm	14.1 (10.5–15.7)*	16.75 (15–17.9)	0.02	0.833
HR	pbm	112.5 (100–166)*	94.5 (73–103)	0.03	0.944
CO	l/min	2.5 (1.9–4.1)	2.35 (1.2–3.6)	0.42	0.222
E	cm/s	57.1 (37.7–69.7)*	44.65 (37–55.7)	0.048	0.778
A	cm/s	54.65 (43.8–57.5)*	39.3 (28.1–51.3)	0.013	1.0
E/A		1.01 (0.8–1.6)	1.25 (0.8–1.5)	0.15	0.667
DecT	ms	99.8 (62–162)*	160.6 (111.7–222)	0.02	0.944
e′ Sep	cm/s	8.52 (5.5–11.3)	8.55 (6.8–9.7)	0.97	0.056
a′ Sep	cm/s	10.1 (5.2–13.8) *	6.5 (4.7–10.5)	0.03	0.833
e′/a′ Sep		0.83 (0.5–1.8) *	1.4 (0.8–1.8)	0.04	0.833
E/e′ Sep		6.25 (5.1–10.4)	5.75 (4.1–6.5)	0.06	0.722
e′ Lat	cm/s	12.4 (9.3–15.2) *	10.6 (6.7–13)	0.03	0.611
a′ Lat	cm/s	10.57 (7–16.6)	9.22 (5.6–11.3)	0.23	0.722
e′/a′ Lat	cm/s	1.4 (0.6–2.1)	1.25 (0.71–1.97)	0.81	0.028
E/e′ Lat	cm/s	4.55 (2.8–7.0)	4.35 (3.4–5.9)	0.83	0.056
LVL	mm	36.10 (29.76–40.14)**	42.01 (40.1–47.2)	0.008	0.889

Values are presented as median (data range). The comparison was done between the obtained measurements in the same individuals at the baseline and 20 min post-xylazine injection (n = 8). ^*^p < 0.05. rc, ranked biserial correlation. EDV, end-diastolic volume; ESV, end-systolic volume; EF, ejection fraction; FS, fractional shortening; LA/Ao, left atrial diameter to aortic diameter ratio; RVOT, right ventricular outflow tract; LVOT, left ventricular outflow tract; HR, heart rate; CO, cardiac output; E, the early diastolic velocity of mitral inflow; A, late diastolic velocity of mitral inflow; E/A early to late mitral inflow ratio; DecT, deceleration time; e′ and a′, annular tissue velocity, Sep, septal, Lat, lateral. LVL, left ventricular length.

### Color M-mode echocardiography for the assessment of IVPD and IVPG

#### Feasibility of imaging and repeatability of CMME indices

**Figure 1:**
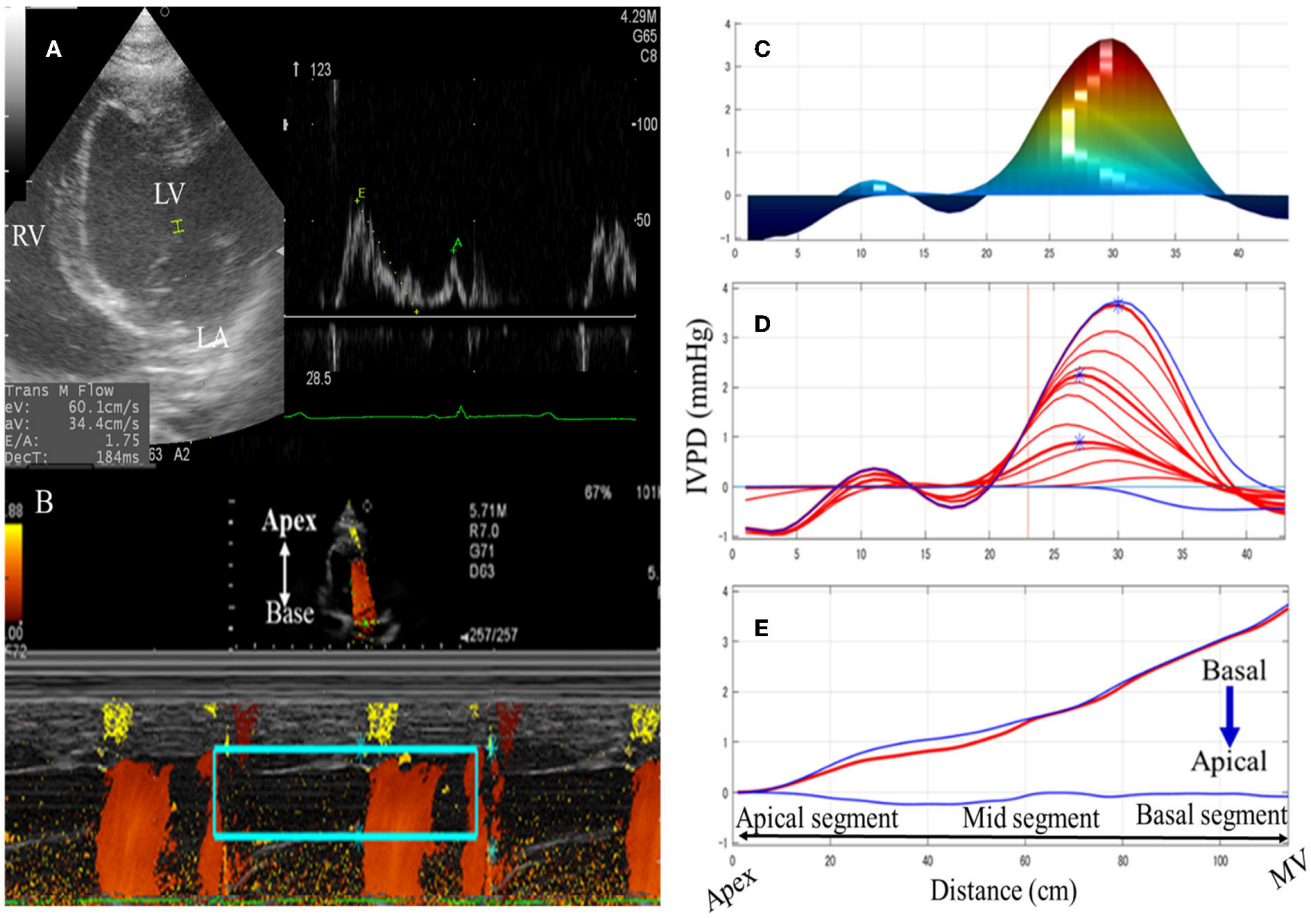
Schematic illustration of color M-mode echocardiographic (CMME) in goats. The left apical four-chamber view was optimized to evaluate the mitral inflow **(A)**. After that, the proper machine setting was started to initiate CMME. IVPG tracing was switched on to trace the entire left ventricular inflow tract from the LV base to the apex. Captured CMME images **(B)** were used for IVPD/IVPG measurements of the area of interest (mitral inflow, rectangular green box) using MATLAB software based on Euler's equation. Measurements of the time from q wave to the start of mitral inflow, from q wave to the peak mitral inflow, and from q wave to the end of aortic flow were separately added to the software's dialog box using ECG displayed on the ultrasound machine. Manual correction of the analysis was limited to noisy photos so that the measurement area matches the IVPD/IVPG position by the software. Temporal and spatial profiles of IVPD **(C)**, and IVPD time distribution **(D,E)** were automatically calculated. MV, mitral valve; LV, left ventricle; LA, left atrium.

The CMME was feasible in all examined goats (100%) (Figure 1). The estimated time to get the entire streamline of the left ventricular inflow from the left atrium to the LV apex across the mitral valve, excluding the MATLAB analysis, was 13 ± 5 min. The IQS of CMME images was done by the qualitative examination of echo- and MATLAB-derived photos. Examination of goats by CMME was excellent (12 goats, 75%) or very good (4 goats, 25%). The LV free wall was not entirely visualized in these four goats; however, the mitral inflow was optimized for CMME. On MATLAB analysis, all goats showed an excellent presentation of the IVPD curves. Overall, the average CMME imaging and software analysis scores obtained by the two observers were 4.2 ± 0.5 and 3.9 ± 0.7, respectively.

**Figure 2:**
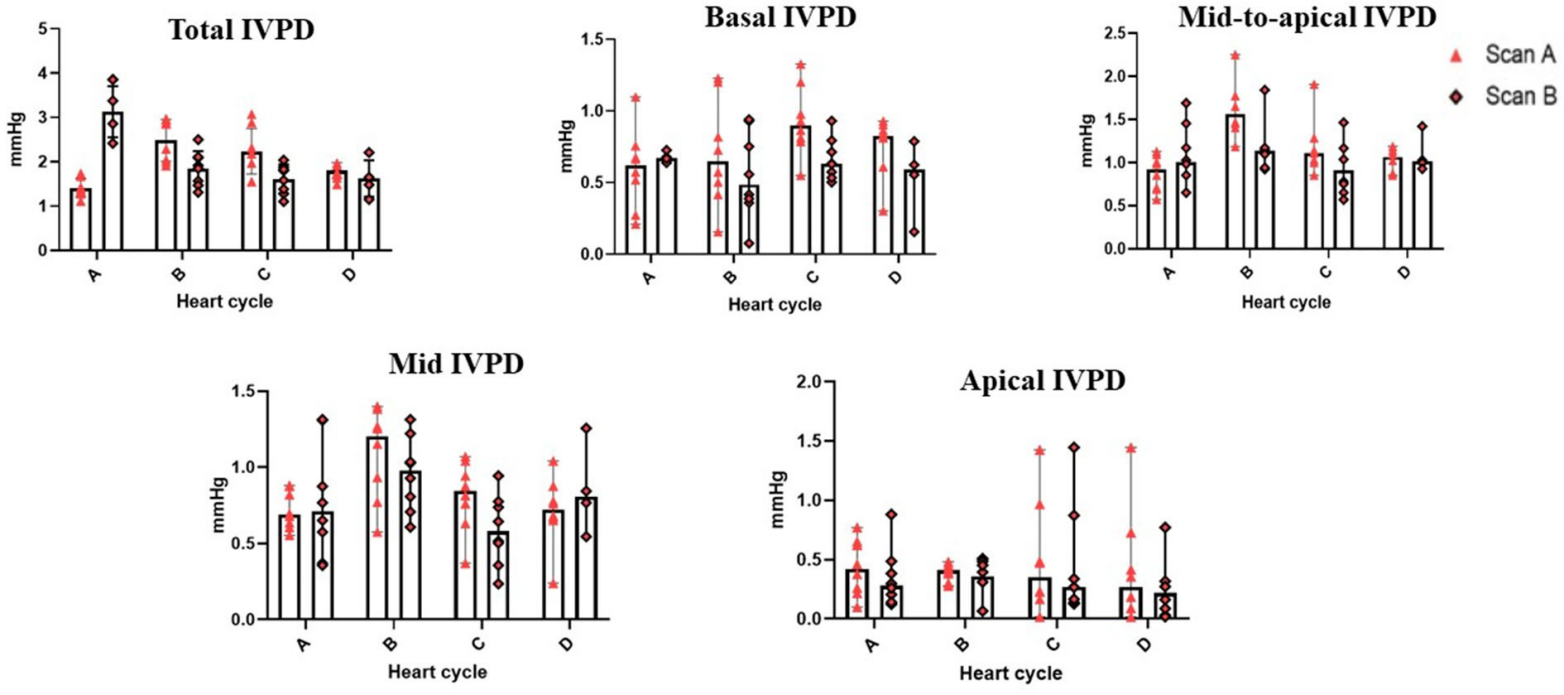
Variability of CMME-derived intraventricular pressure difference (IVPD). Variability was calculated within goats between heart cycles (four heart cycles were selected) and between time by two observers (scans A and B); *n* = 8.

**Figure 3:**
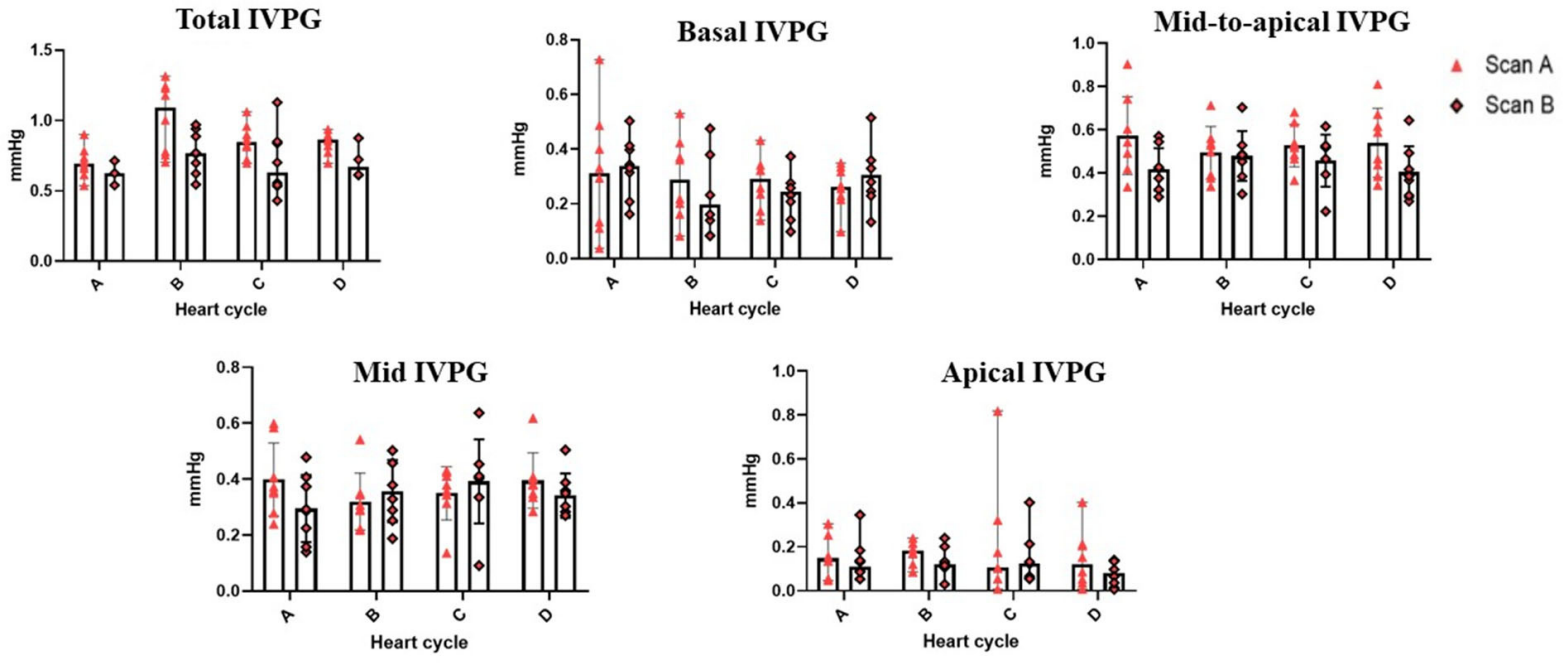
Variability of CMME-derived intraventricular pressure gradients (IVPG). Variability was calculated within goats between heart cycles and between time by two observers (scans A and B); *n* = 8.

Table 2 summarizes the repeatability of CMME indices. The intraobserver variability of all CMME data displayed low to moderate variability considering different heart cycles (95% CV: 11.96–23.9) except for apical IVPD and apical IVPG which showed high variability (CV = 31.81, 29.5), respectively. Between observers, the data revealed low to moderate variability (95% CV range: 4.11–23.67). By two-way ANOVA, the goat factor, the time factor, as well as the goat X time interaction showed no significant effect on the obtained IVPD and IVPG measurements (Figures 2, 3). Within goats, there were no significant differences in all measurements. The average ICC for the obtained data was acceptable in all measurements (ICC > 0.75) except for apical IVPD and apical IVPG (ICC = 0.601, 0.698), respectively.

**Table 2:** Repeatability of color M-mode echocardiographic measurements in adult healthy male Shiba goats.

**Variables**	**Mean±SD**	**95% CI of mean (L-U)**	**95 % CV**		**ICC**
			**Between cycles**	**Between time**	
Total IVPD	2.02 ± 0.41	1.55–2.49	20.24	23.04	0.91
Basal IVPD	0.67 ± 0.14	0.55–0.80	20.54	11.63	0.80
Mid-to-apical IVPD	1.14 ± 0.24	0.91–1.36	21.29	13.66	0.94
Mid IVPD	0.82 ± 0.17	0.66–0.97	20.52	14.11	0.94
Apical IVPD	0.32 ± 0.10	0.24–0.41	31.81	17.27	0.70
Total IVPG	0.78 ± 0.13	0.67–0.88	16.22	4.72	0.82
Basal IVPG	0.29 ± 0.07	0.24–0.35	23.90	12.22	0.81
Mid-to-apical IVPG	0.49 ± 0.06	0.44–0.53	11.69	6.81	0.77
Mid IVPG	0.36 ± 0.06	0.30–0.41	17.95	10.99	0.70
Apical IVPG	0.15 ± 0.04	0.11–0.18	29.52	19.66	0.69

Color M-mode echocardiography-derived variables in goats (n = 8). CV, coefficient of variation; CI, confidence interval; ICC, intraclass correlation coefficient. All variables are measured by mmHg.

#### Effect of sedation on IVPG and IVPD analysis

**Figure 4:**
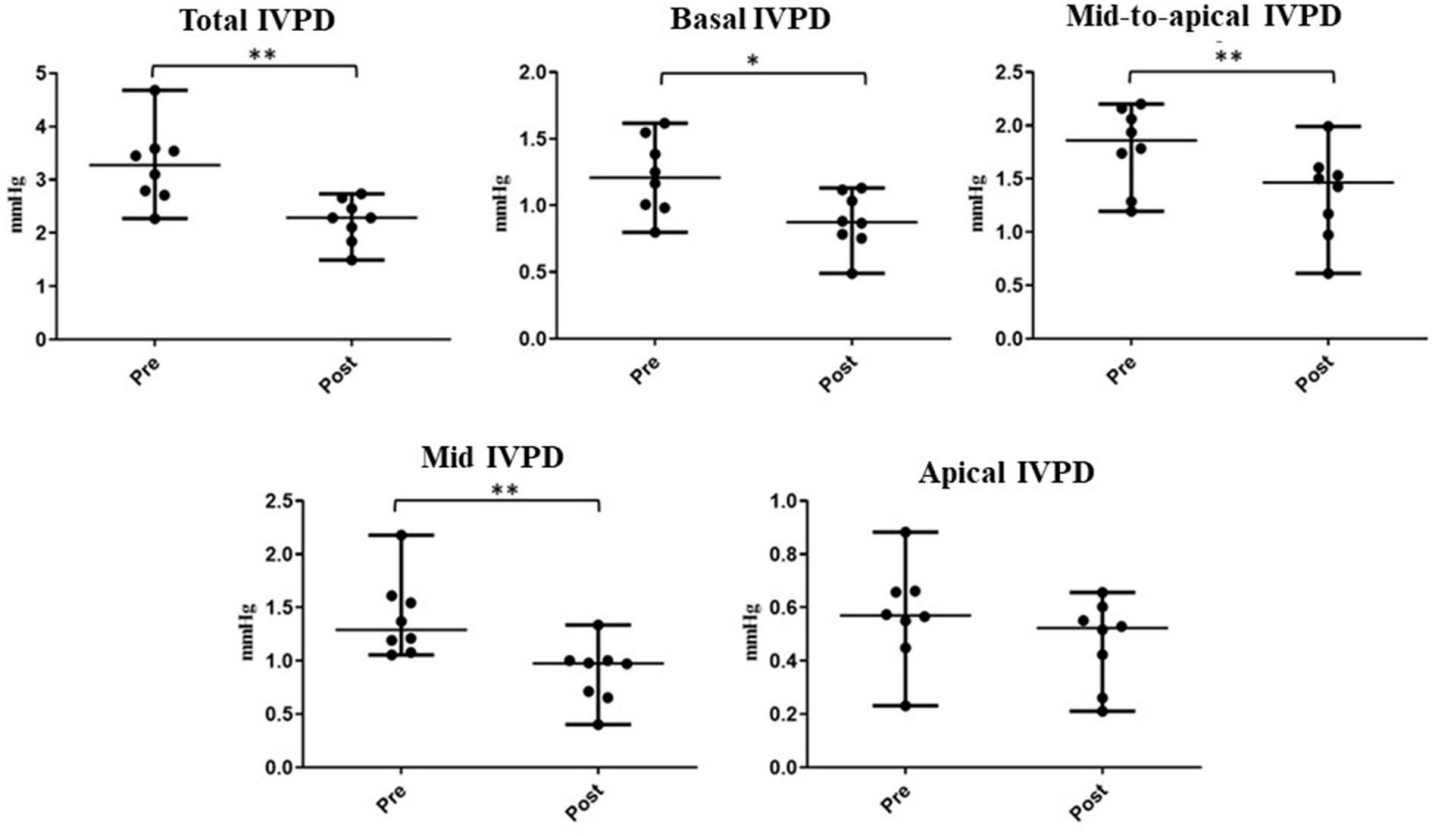
Changes in IVPD indices measured by color M-mode echocardiography before and after sedation with xylazine in goats (*n* = 8). Plots showing the median (central horizontal line) and range (upper and lower lines). *p < 0.05, **p < 0.01.

**Figure 5:**
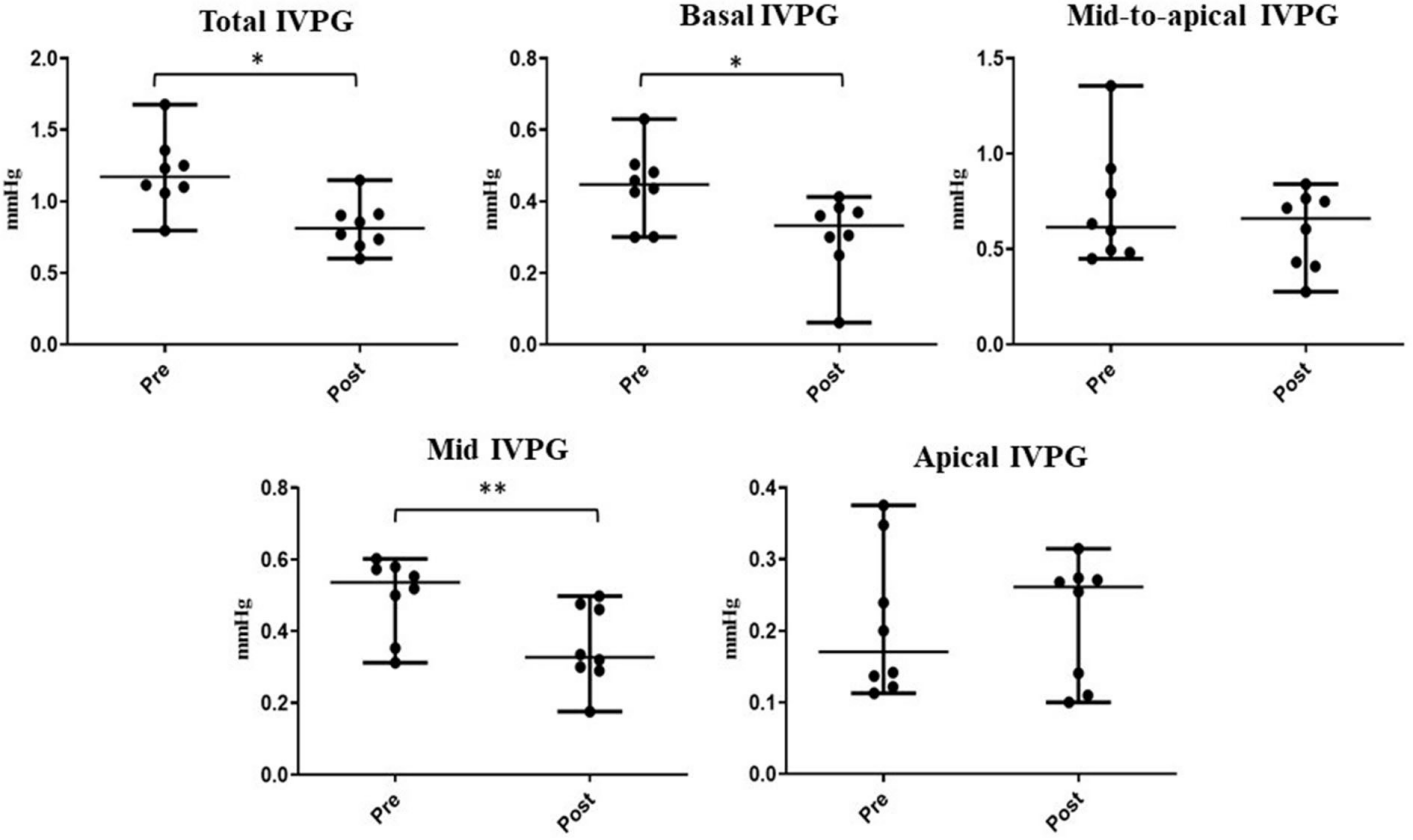
Changes in IVPG indices before and after sedation with xylazine in goats (*n* = 8). Plots showing the median (central horizontal line) and range (upper and lower lines). *p < 0.05, **p < 0.01.

The time required to perform CMME, excluding the MATLAB analysis, was shorter after sedation (range: 8–14 min) compared with that at the baseline (range: 10–17 min). Regarding the IVPD (Figure 4), the data revealed a significant reduction in total IVPD (median: 3.28 vs. 2.29; *P* = 0.005), basal IVPD (median: 1.21 vs. 0.87; *P* = 0.023), mid-to-apical IVPD (median: 1.86 vs. 1.47; *P* = 0.008), and mid-IVPD (median: 1.29 vs. 0.98; *P* = 0.008) after xylazine administration compared with their values at the baseline. In the same way, significant reduction in total IVPG (median: 1.17 vs. 0.81; *P* = 0.016), basal IVPG (median: 0.45 vs. 0.33; *P* = 0.015), and mid-IVPG (median: 0.54 vs. 0.33; *P* = 0.008) was also observed post-xylazine administration (Figure 5). Meanwhile, apical IVPD, mid-to-apical IVPG, and apical IVPG showed no significant changes (*P* > 0.05). The difference between baseline and post-xylazine measurements was clinically relevant and associated with a large effect size on total IVPD, basal IVPD, mid-to-apical IVPD, and mid-IVPD (rc: 1.0, 0.889, 1.0, 1.0, respectively) as well as total IVPG, basal IVPG, and mid IVPG (rc: 0.94, 1.0, 1.0, respectively).

#### Correlation between IVPD/IVPG and conventional echocardiographic variables

Table 3 shows Spearman's correlation between IVPD/IVPG indices and conventional echocardiographic measurements. Total IVPD was positively correlated with HR (r_s_ = 0.615, *P* = 0.013) and negatively correlated with LV length (r_s_ = −0.506, *P* = 0.046). Mid IVPD found a significant positive correlation with lateral annular velocity a′ (r_s_ = 0.567, *P* = 0.022); meanwhile, basal IVPD was positively correlated with lateral annular e′ (r_s_ = 0.539, *P* = 0.031). The apical IVPD showed a positive correlation with HR, mitral A velocity, and septal a′ velocity (r_s_ = 0.521, 0.724, 0.512; *P* = 0.041, 0.002, 0.043, respectively) and negative correlation with E/A ratio and deceleration time (r_s_ = −0.601, −0.509, *P* = 0.014, 0.044, respectively). Moreover, total IVPG showed a positive correlation with HR and mitral A velocity (r_s_ = 0.675, 0.671; *P* = 0.005, 0.004, respectively) and significant adverse correlation with E/A ratio and deceleration time (r_s_ = −0.736, −0.600; *P* = 0.001, 0.014, respectively). Mid-to-apical IVPG showed a significant positive correlation with annular lateral e′ (r_s_ = 0.514, *P* = 0.042). Moreover, the relationship between total IVPD and A velocity; basal IVPD and HR; apical IVPD and LV length; total IVPG and LV length; mid IVPG and HR, mitral A velocity and E/A; as well as the relationship among mid-to-apical IVPG and HR, mitral A velocity, and E/A ratio were significant (*P* < 0.10).

**Table 3:** Correlation between CMME indices and conventional echocardiographic measurements in goats.

**Variables**	**IVPD**										**IVPG**									
	**Total**		**Basal**		**Mid-to-apical**		**Mid**		**Apical**		**Total**		**Basal**		**Mid-to-apical**		**Mid**		**Apical**	
	**r_s_**	***P***	**r_s_**	***P***	**r_s_**	***P***	**r_s_**	***P***	**r_s_**	***P***	**r_s_**	***P***	**r_s_**	***P***	**r_s_**	***P***	**rs**	***P***	**r_s_**	***P***
HR	0.615*	0.013	0.472	0.066	0.266	0.316	0.135	0.615	0.521*	0.041	0.675*	0.005	−0.009	0.976	0.492	0.055	0.492	0.055	−0.078	0.773
EDV	−0.115	0.672	−0.250	0.349	−0.103	0.705	0.068	0.805	−0.129	0.633	−0.169	0.528	0.238	0.373	−0.150	0.579	−0.150	0.579	0.121	0.656
E	0.226	0.399	0.256	0.339	0.141	0.602	−0.147	0.587	0.229	0.393	0.011	0.700	−0.371	0.158	0.100	0.713	0.100	0.713	−0.409	0.116
A	0.453	0.078	0.297	0.264	0.176	0.513	−0.038	0.888	0.724*	0.002	0.671*	0.004	−0.124	0.649	0.432	0.094	0.432	0.094	−0.332	0.209
E/A	−0.219	0.416	−0.018	0.947	0.078	0.774	0.138	0.611	−0.601*	0.014	−0.736*	0.001	−0.133	0.623	−0.456	0.076	−0.456	0.076	−0.012	0.965
DecT	−0.326	0.217	−0.076	0.778	0.088	0.745	0.168	0.535	−0.509*	0.044	−0.600*	0.014	0.226	0.399	−0.221	0.412	−0.221	0.412	0.306	0.249
e′ Sep	−0.165	0.542	−0.330	0.212	−0.159	0.556	−0.156	0.564	0.180	0.506	−0.043	0.873	−0.161	0.553	0.156	0.564	0.156	0.564	−0.082	0.761
a′ Sep	0.168	0.535	−0.149	0.583	0.202	0.454	0.162	0.549	0.512*	0.043	0.406	0.119	0.090	0.741	0.241	0.368	0.241	0.368	−0.091	0.737
e′/a′ Sep	−0.283	0.289	−0.034	0.901	−0.287	0.281	−0.265	0.322	−0.396	0.128	−0.412	0.113	−0.176	0.514	−0.172	0.525	−0.172	0.525	0.016	0.952
E/e′ Sep	0.279	0.296	0.404	0.121	0.311	0.241	0.202	0.453	0.050	0.854	−0.163	0.546	−0.158	0.560	−0.156	0.563	−0.156	0.563	−0.307	0.248
e′ Lat	0.361	0.170	0.539*	0.031	0.277	0.300	0.246	0.359	0.196	0.468	0.395	0.130	0.411	0.114	0.514*	0.042	0.514	0.042	0.212	0.431
a′ Lat	0.373	0.155	0.090	0.741	0.409	0.115	0.567*	0.022	0.343	0.193	0.064	0.814	0.274	0.305	0.031	0.910	0.031	0.910	0.243	0.365
e′/a′ Lat	0.027	0.922	0.251	0.349	−0.093	0.732	−0.181	0.502	−0.178	0.509	0.161	0.552	0.111	0.684	0.318	0.230	0.318	0.230	0.085	0.753
E/e′ Lat	−0.150	0.579	−0.296	0.266	−0.050	0.854	−0.121	0.656	−0.004	0.987	−0.362	0.168	−0.362	0.168	−0.281	0.292	−0.281	0.292	−0.322	0.223
LVL	−0.506*	0.046	−0.224	0.405	−0.226	0.399	−0.141	0.602	−0.438	0.090	−0.452	0.079	0.000	0.901	−0.265	0.322	−0.265	0.322	0.012	0.966

Spearman's correlation (r_s_) between IVPD and IVPG indices and conventional echocardiographic measurements in goats (n = 8). *p < 0.05.

## Discussion

The establishment of new technology regarding cardiac function evaluation in ruminants is a point of interest for both clinical and research purposes (24, 37, 38). In this study, we examined the use of the novel CMME-specific variables (IVPD and IVPG) in goats. Furthermore, we reported the normal values of IVPG and IVPD indices and explored the effect of sedation by xylazine on the obtained measurements.

Visualization of different cardiac windows from standard views was optimized in all goats. In the current study, systolic function indices (FS% and EF%) showed no significant changes between pre- and post-xylazine administration although the heart function was significantly reduced (39). Pulsed TDI was used to evaluate the regional wall velocity at both sides of mitral valve attachment (40, 41). TDI at the septal and posterior walls yield two negative diastolic velocities (e′, a′) which are representative of myocardial relaxation and atrial contraction, respectively (42). For the interpretation of the diastolic cardiac function, the annular velocity e′ wave is considered an indicator for relaxation and elastic recoil of the LV; meanwhile, the E/e′ ratio is crucial for the diagnosis of congestion (41).

In our study, diastolic function measurements were not homogenous based on conventional echocardiography examination. In this regard, the E/A ratio did not significantly change after xylazine administration, and the tissue Doppler velocities showed variations at the septal and free walls of the LV walls. Thus, septal a′ velocity, lateral e′ velocity, and septal E/e′ were significantly reduced after sedation. In contrast, the velocity of septal e′, lateral a′, and E/e′ of the free wall did not significantly change. The assessment of mitral inflow and myocardial tissue velocity is frequently used to evaluate diastolic function. Nevertheless, accurate diagnosis of diastolic dysfunction is a controversial issue that requires a combination of different diagnostic approaches (2, 4).

Recent studies revealed that CMME-derived IVPD/IVPG is significantly related to the *Tau* (a reliable index of left ventricular diastolic function) (43), and hence could non-invasively evaluate the diastolic function (1, 12). To our knowledge, the current study is the first to investigate the validity of IVPD and IVPG in goats. The left parasternal long axis four-chamber view was optimized for adequate Doppler alignment using CMME as previously described in other animals (8, 21). The imaging quality and software analysis scores for CMME obtained in the current study were acceptable. All CMME variables, except apical IVPD and apical IVPG, showed acceptable repeatability with low to moderate variability. These results are quite reasonable particularly when we consider that these data were calculated before sedation. The higher CV of apical indices may be related to variation in the color resolution of the apical part of CMME images compared with the basal and mid parts (7). Therefore, CMME can be used for further evaluation of heart function in goats and the apical variables should be considered with caution.

The present study also intended to explore the agreement of the IVPG/IVPD findings with other studies conducted in dogs, the largest animal species in which CMME was previously studied. As previously described, MATLAB-specific software (MathWorks) classifies the IVPD/IVPG into basal, mid-to-apical, mid, and apical parts (1, 8, 21). In the current study, the reported IVPD and IVPG ranges were comparable to recently published studies on anesthetized Beagle dogs under various loading conditions (8), during chemotherapy treatment (1), and non-anesthetized client-owned dogs (21). In the later study (*n* = 58, BW 1.3–42.3 kg), this classification yielded 0.92 ± 2.86, 0.39 ± 1.13, 0.47 ± 1.41 and 0.25 ± 0.33 for total, basal, mid, and apical IVPD, respectively, and 0.30 ± 0.94, 0.12 ± 0.36, 0.14 ± 0.44 and 0.11 ± 0.09 for total, basal, mid and apical IVPG parts (21). Another study in Beagle dogs yielded 0.12 ± 0.86, 0.09 ± 0.35, 0.11 ± 0.51, 0.08 ± 0.48 and 0.03 ± 0.03 for total, basal, mid-to-apical, mid, and apical IVPG parts (1). Our study elected not to focus only on normal goats but to investigate the CMME in goats after an alteration of heart function. This was different than the other published studies on IVPG in dogs since the net effect of sedation or anesthesia on CMME indices in dogs has not been reported. Despite the difference in species and body weight between our study and other canine studies (1, 8), there was an acceptable similarity in the obtained CMME measurements in dogs and goats. However, the study sample in our study had subjectively fewer goats and was treated with xylazine when compared to case numbers and conditions in the previous studies. Overall, this indicates that the CMME-derived IVPD/IVPG is comparatively acceptable in goats as another species to study this novel echocardiographic approach. Moreover, our findings suggested that the CMME technique may be possible to use in other farm animals for further physiological and pharmacological studies.

In ruminants, xylazine is used for pre-anesthesia and induction. The safety margin of xylazine is comparatively narrow which could induce rapid collapse in animals if the dose is not properly adjusted (44). In the current study, as in previous reports, xylazine administration reduced HR and Doppler measurements of the pulmonary artery, and aorta as well as the mitral inflow (E and A waves) from the baseline. These reductions in the pressure, heart rate and cardiac contraction, and elevation in afterload are caused by its chronotropic properties (39, 45, 46).

In this study, xylazine was selected as a simple medicine to induce alteration in the heart function for subsequent evaluation of the response of IVPG/IVPD to the hemodynamic changes in goats. In this regard, xylazine administration resulted in significantly reduced IVPD and IVPG indices; meanwhile, the E/A ratio was not significantly changed. This indicates the potential importance of IVPG and IVPD to detect preload changes as confirmed by a significant reduction in E and A waves of the mitral inflow. The importance of IVPG and IVPD as indicators of diastolic function has been demonstrated (16). It has been evidenced that each IVPD or IVPG part could reflect a specific function during heart function evaluation. For example, basal IVPD is positively related to the increased volume load in case of congestion (16, 33), advanced diastolic dysfunction reduce mid IVPG (33, 42), left ventricular active relaxation correlated well with mid-to-apical IVPG, and apical IVPG is related to the active power of blood withdrawal from the left atrium by LV (11, 15). In our study, changes in IVPD and IVPG indices could be ascribed to reduced contractility and blood flow and increased LV length after xylazine injection.

In the current study, xylazine administration exerted a profound effect on atrial contractility as observed by reduced late mitral velocity (A, rc = 1.0) and a annular tissue velocity values. However, the E/e' ratio (left atrial pressure indicator), which was measured at both septal and lateral mitral annulus (47), was not significantly changed. Previous studies reported that the evaluation of left atrial pressure using E/e' has certain limitations and basal IVPG could be more suitable for the monitoring of the preload in response to medication. In this regard, a previous study (16) reported inconsistent results of TDI in loading changes and found only that septum E/e' was significantly increased after colloidal solution infusion in contrast to the IVPG which was significantly responding to the infusion. In the present work, apical IVPD and total IVPG were negatively and significantly correlated with late mitral inflow (A) wave velocity, E/A ratio, and deceleration time of the mitral inflow. The presence of significant correlation between CMME indices and conventional parameters might enforce the usefulness of CMME-derived IVPG/IVPD to evaluate heart function especially diastolic one away from invasive procedures. However, proper interpretation of these relationships should be cautiously considered because of small sample size and short-term evaluation.

In this study, the LV length was slightly increased after xylazine administration. However, only total IVPD was negatively correlated with the LV length. This was in agreement with a previous study in sheep which showed a linear relationship between the decrease in intraventricular pressure and LV length (48). In contrast, the study by Popović et al. (34) revealed that LV length was positively correlated with IVPD but not associated with IVPG. In the latter study, the authors investigated different species with different heart sizes. However, upon the examination of their results, we found that the goat was almost close to the dog and human values that almost show a negative relationship of LV length with IVPG values in contrast to smaller heart size species. IVPD was also reported to be related to the LV length in dogs (21), but this study used many dogs in which small breed dogs were predominant. IVPG is calculated from IVPD to exclude the effect of the LV length. Although both IVPD and IVPG approximately showed a similar trend of results before and after xylazine medication, IVPG will be more reliable when there is a great variation in LV length, or when the LV length significantly changes due to medication as in the current study. However, a combination between IVPD and IVPG may be better to explore the actual pressure difference and the pressure slope in the LV. Our results revealed positively correlated HR with total IVPD and apical IVPD, as well as total IVPG. During restraint of animals, the HR can be increased, and subsequently, the pressure increases (49), and the reverse is true after medication with xylazine.

Cardiac tissue damages in ruminants due to various infectious diseases and nutritional disorders are frequently occurring without expressing clear clinical signs in the early stage but they rather can be detected in the late stages of cardiac diseases or during necropsy (50). IVPD and IVPG assessments are useful in the early detection of cardiac dysfunction in animal models, such as dogs and rats. In farm animals too, especially goats, IVPD and IVPG could deepen the understanding of the pathophysiological and pharmacological aspects of intraventricular flow. Also, clinical trials are warranted to evaluate whether this technique helps detect cardiac pathologies earlier to decide whether to treat or cull those animals.

### Limitations

The present study includes only adult male goats with small sample size. The number of goats in Japan has a limited distribution since goats are infrequently accepted as food for humans and are mostly used for research and educational purposes by elementary students (51, 52). Male goats were selected to avoid the biased results from female reproductive cycles that should be addressed in another study. Simultaneous assessment of IVPD/IVPG using invasive catheterization and CMME was not provided.

## Conclusion

To the best of the author's knowledge, this is the first study of the quantitative measurement of the IVPD and IVPG in goats using a novel CMME technique. The CMME-derived IVPD and IVPG are valid methods in awake and sedated goats. A translational lens of this novel study, in conjunction with conventional echocardiographic methods, might deepen understanding of the LV hemodynamics in goats as well as other farm animals for further experimental and clinical studies.

## Data availability statement

The raw data supporting the conclusions of this article will be made available by the authors, without undue reservation.

## Ethics statement

The animal study was reviewed and approved by the Ethical Committee of the Animal Medical Center, Tokyo University of Agriculture and Technology, and Technology (Ethical No: 30–78).

## Author contributions

ASM and RT: experiment design. ASM, HS, TY, and KM: echocardiography. ASM, HS, TY, and ME: investigation. ASM, TY, HS, KS, HAA, KT, KM, LH, and AU: software and data analysis. ASM, LH, and KS: data collection. ASM and HAA: writing and drafting. HS, ME, LH, KT, and GW: critical editing. GW and RT: supervision. All authors reviewed and edited the final version. All authors contributed to the article and approved the submitted version.

## Conflict of interest

The authors declare that the research was conducted in the absence of any commercial or financial relationships that could be construed as a potential conflict of interest.

## Publisher's note

All claims expressed in this article are solely those of the authors and do not necessarily represent those of their affiliated organizations, or those of the publisher, the editors and the reviewers. Any product that may be evaluated in this article, or claim that may be made by its manufacturer, is not guaranteed or endorsed by the publisher.
